# Fucoidan attenuates radioiodine-induced salivary gland dysfunction in mice

**DOI:** 10.1186/s12903-019-0894-2

**Published:** 2019-08-30

**Authors:** Young-Mo Kim, Jeong Mi Kim, Ji Won Kim, Mi Eun Choi, Seok-Ki Kim, Jeong-Seok Choi

**Affiliations:** 1 Translational Research Center, Inha University, Incheon, Republic of Korea; 2 Department of Otorhinolaryngology-Head and Neck Surgery, Inha University College of Medicine, 27, Inhang-ro, Jung-gu, Incheon, 22332 Republic of Korea; 3 Department of Nuclear Medicine, National Cancer Center, Goyang, Republic of Korea

**Keywords:** Radioiodine, Salivary gland, Fucoidan, Antioxidant, Thyroid cancer, Animal model

## Abstract

**Background:**

Radioiodine (RI) treatments can destroy the cellular components of salivary glands (SG) and disrupt their function. This study investigated whether fucoidan could attenuate radioiodine-induced SG dysfunction in a mouse model.

**Methods:**

Female C57BL/6 mice (*n* = 36) were classified into three groups; i) a normal (control) group, ii) an RI-treated group (0.2 mCi/20 g mouse, administered orally), and iii) a fucoidan and RI-treated group. Mice in each group were classified into three subgroups and sacrificed at 2, 4, and 12 weeks after RI treatment. The measurements of salivary flow rates and lag times and histomorphologic examinations were performed, and apoptotic assays were conducted. Changes in salivary ^99m^Technetium (Tc)-pertechnetate parameters using single-photon emission computed tomography were followed.

**Results:**

Salivary flow rates and lag times in the fucoidan group were improved compared to the RI-treated group. Histologic examinations of SGs in the fucoidan group showed mucin-rich parenchymal areas and reduced periductal fibrosis as compared to the RI-treated group. Moreover, compared with the RI-treated group, fucoidan-treated groups showed evidence of cytoprotection, with a greater number of salivary epithelial cells and myoepithelial cells being observed. Fewer apoptotic cells were observed in the fucoidan group as compared to the RI group. The extent of ^99m^Tc pertechnetate excretion in the fucoidan group was similar to that of the control group.

**Conclusion:**

Our results demonstrate that fucoidan administration before RI treatment could attenuate RI-induced SG damage and provides a possible candidate for preventing SG damage induced by RI.

## Background

In the treatment of thyroid cancer, radioiodine (RI) administration is essential to remove the remaining cancerous tissue and prevent cancer recurrence. Unfortunately, RI not only affects thyroid tissue, but also enters into other tissues such as the salivary glands (SGs), breasts, and gastrointestinal tract, and causes unwanted side effects. The most common side effect of RI is the deterioration of the SGs, which causes salivary pain and swelling. Later, RI intake leads to xerostomia, oral discomfort, taste disorders, and difficulty with food ingestion [1]. RI treatment may not only cause discomfort in patients but may also interfere with the treatment of thyroid cancer. In this respect, it is important to manage and control the function of the SGs [2].

The ionizing radiation caused by RI induces the production of reactive oxygen species (ROS), which destroys cellular components in normal tissue. In order to eliminate the action of ROS, various intracellular antioxidants exist in the cells to scavenge ROS and lessen cellular oxidative stress [3]. Although amifostine is known as the only radio-protective drug by scavenging ROS, it has various side effects and is quite costly. Also, studies on amifostine associated with RI therapy have been limited [4]. Various studies have attempted to rehabilitate SGs exposed to radiation. These attempts include cell therapy, using mitigators such as growth hormones, and using radioprotectors such as antioxidants [5]. Moreover, various studies have been attempted to prevent RI-induced SG damage with antioxidants [6, 7].

Fucoidan is a sulfated polysaccharide found in brown seaweeds and has various biological activities, including antioxidant, anti-inflammatory, and immunomodulatory activities [8–11]. The effect of fucoidan on the restoration of SG dysfunction induced by RI administration has not been described in the literature. The aim of this study was to investigate whether fucoidan administered before RI therapy could attenuate RI-induced SG dysfunction morphometrically and functionally in a mouse model.

## Methods

### Animal studies

Thirty-six mice (4-week-old female C57BL/6; 18–22 g) were purchased from Research Model Producing Center Co. Ltd. (Orient Bio, Seongnam, Korea) and housed according to the Guideline for the Care and Use of Laboratory Animals from the National Cancer Center, Korea. Animals were divided into the following three groups (*n* = 12 per group): normal control group, RI-treated group (oral administration of 0.01 mCi/g body weight, ^131^I; New Korea Industrial, Seoul, Korea), fucoidan (intraperitoneal [i.p.] administration of 10 mg/kg, twice 6 and 0.5 h before RI exposure; Santa Cruz, CA, USA,) + RI-treated group. Each group was subdivided in three subgroups of 4 animals based on time of sacrifice (2, 4, and 12 weeks post-RI). For maintaining a euthyroid state, all mice were given 1.5 μg thyroxine (T4)/100 g body weight and 1% calcium lactate in their drinking water. All animal studies were conducted using protocols approved by the Institutional Animal Care and Use Committee.

### Measurements of body weights and SG functions

After anesthetizing a mouse using ketamine (100 mg/kg) and xylazine (5 mg/kg), each mouse was weighed. Pilocarpine was dissolved in phosphate-buffered saline (PBS; 0.5 mg/mL) and administered i.p. to mice (0.01 mL/g body weight). Total saliva was collected from the oral cavity of each mouse for 10 min in pre-weighed tubes after pilocarpine administration using a specifically designed suction device. Lag times (the time saliva begins to appear after pilocarpine administration) were also measured. After saliva collection, the mice were sacrificed by the cervical dislocation and submaxillary glands were collected.

### Morphological analysis of tissues and TUNEL assay

Tissues obtained from the submaxillary glands were fixed in 4% paraformaldehyde, embedded in paraffin, and sectioned. SG sections were stained with alcian blue (AB) and Masson’s trichrome (MT). Immunostaining was performed using standard procedures. The tissue slides were incubated with rabbit polyclonal antibodies as follows: anti-AQP5 (aquaporins 5, diluted 1:200; Alomone Labs, Jerusalem, Israel), and anti-α-SMA (α-smooth muscle actin, diluted 1:200; Santa Cruz, California, USA). The staining reaction was performed using an LSAB kit (Dako, Carpinteria, CA, USA). Three sections were made for each gland and at least 5 fields per section were observed and evaluated using ImageJ Software to quantify the data.

Apoptosis in the submaxillary glands was determined using a terminal deoxynucleotidyl transferase biotin-dUDP nick-end labeling (TUNEL) assay with an ApopTag Plus in situ Apoptosis Kit (Chemicon Int., Temecula, CA, USA). The number of TUNEL-positive cells was counted in 12 random high power fields.

### Single photon emission computed tomography (SPECT) and SPECT image analysis

At 12 weeks post-RI, whole-body SPECT imaging was conducted and analyzed after ^99m^Tc (technetium) pertechnetate injection. It was performed in the same way as the previous experiment [12, 13].

### Statistical analysis

Kruskal-Wallis test, followed by the post-hoc Dunn’s test was conducted by Graph Pad Prism 5 package (GraphPad Software Inc., La Jolla, CA, USA). Statistical significance was accepted if the *p* value was less than 0.05.

## Results

### Changes in body and salivary gland weight

**Fig. 1:**
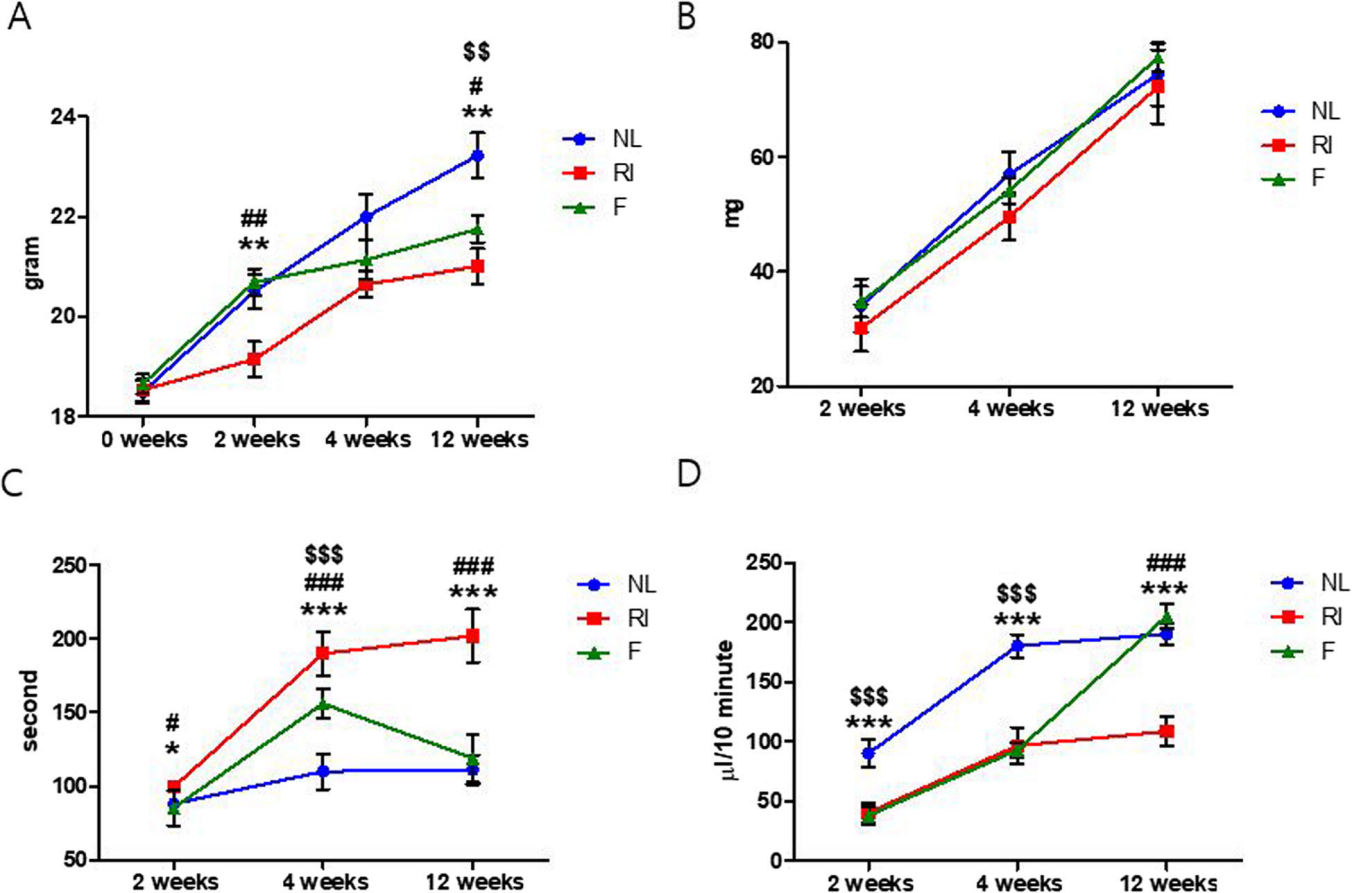
Morphological and functional changes in salivary glands after fucoidan treatment. **a** Body weight, (**b**) glandular weight, (**c**) salivary lag times, and (**d**) flow rates were measured. The mice in the fucoidan groups were heavier than those in the radioiodine (RI)-exposed group (RI group). The weights of the glands were not significantly different between all groups. Lag times and salivary flow rates in the fucoidan group were shorter and greater, respectively, than those in the RI group. Statistical analyses were done using the Kruskal-Wallis test and the Dunn’s post hoc multiple comparison test; ^*^, compared between the normal control and the RI groups; ^#^, compared between the RI and the fucoidan groups; ^$^, compared between the normal control and the fucoidan groups. *, *p* < 0.05; **, *p* < 0.01; ***, *p* < 0.001, #, *p* < 0.05; ##, *p* < 0.01; ###, *p* < 0.001, $$, *p* < 0.01; $$$, *p* < 0.001, *n* = 4 mice in all groups. NL, normal control; RI, RI-exposed group; F, administration of fucoidan before RI exposure

Before the initiation of the experiment, there were no significant differences in body weight among the groups. However, after experimentation, mice in the RI group (18.5 ± 0.2, 19.2 ± 0.3, 20.7 ± 0.3, 21.0 ± 0.4; mean ± standard deviation (SD) at 0, 2, 4, 12 weeks respectively) weighed significantly less than mice in the normal control group (18.5 ± 0.2, 20.5 ± 0.3, 22.0 ± 0.5, 23.2 ± 0.5; mean ± SD at 0, 2, 4, 12 weeks respectively), and mice in the fucoidan group (18.7 ± 0.2, 20.7 ± 0.3, 21.1 ± 0.4, 21.8 ± 0.3; mean ± SD at 0, 2, 4, 12 weeks respectively) weighed significantly more than mice in the RI group at 2 and 12 weeks post-treatment (Fig. 1a, *p* < 0.05). The RI group (30.2 ± 4.1, 49.6 ± 4.2, 72.2 ± 6.4; mean ± SD at 2, 4, 12 weeks respectively) had a tendency to have a lower gland weight compared to normal group (34.1 ± 4.6, 56.9 ± 3.9, 74.4 ± 5.4; mean ± SD at 2, 4, 12 weeks respectively) and fucoidan group (34.8 ± 2.7, 54.1 ± 2.2, 77.3 ± 2.5; mean ± SD at 2, 4, 12 weeks respectively) at 2, 4, and 12 weeks post-RI, but the differences were not statistically significant (Fig. 1b, *p* > 0.05).

### Salivary lag times and salivary flow rates

Significant intergroup differences in lag times were observed at 2, 4, and 12 weeks post-RI administration (Fig. 1c, *p* < 0.05). Lag times in the normal group (88.0 ± 3.0, 110.0 ± 12.0, 111.0 ± 9.9; mean ± SD at 2, 4, 12 weeks respectively) were lower than those of RI group (99.5 ± 2.5, 190.0 ± 15.0, 202.0 ± 18.1; mean ± SD at 2, 4, 12 weeks respectively) at 2, 4, and 12 weeks. At 4 and 12 weeks post-RI, lag times in the fucoidan-treated group (85.3 ± 11.7, 156.0 ± 10.1, 119.0 ± 16.1; mean ± SD at 2, 4, 12 weeks respectively) were significantly shorter than in the RI group. Also, salivary flow rates in the normal group (90.3 ± 11.4, 180.6 ± 9.6, 190.9 ± 8.9; mean ± SD at 2, 4, 12 weeks respectively) were higher than those of RI group (40.5 ± 8.2, 96.7 ± 15.5, 108.9 ± 12.2; mean ± SD at 2, 4, 12 weeks respectively) and fucoidan group (38.4 ± 7.6, 93.2 ± 6.1, 205.6 ± 9.8; mean ± SD at 2, 4, 12 weeks respectively) at 2 and 12 weeks post-treatment (Fig. 1d, *p* < 0.05). At 12 weeks post-RI, salivary flow rates in the fucoidan-treated group returned to a similar level to the normal group (Fig. 1d, *p* < 0.05).

### Histological changes and apoptosis

**Fig. 2:**
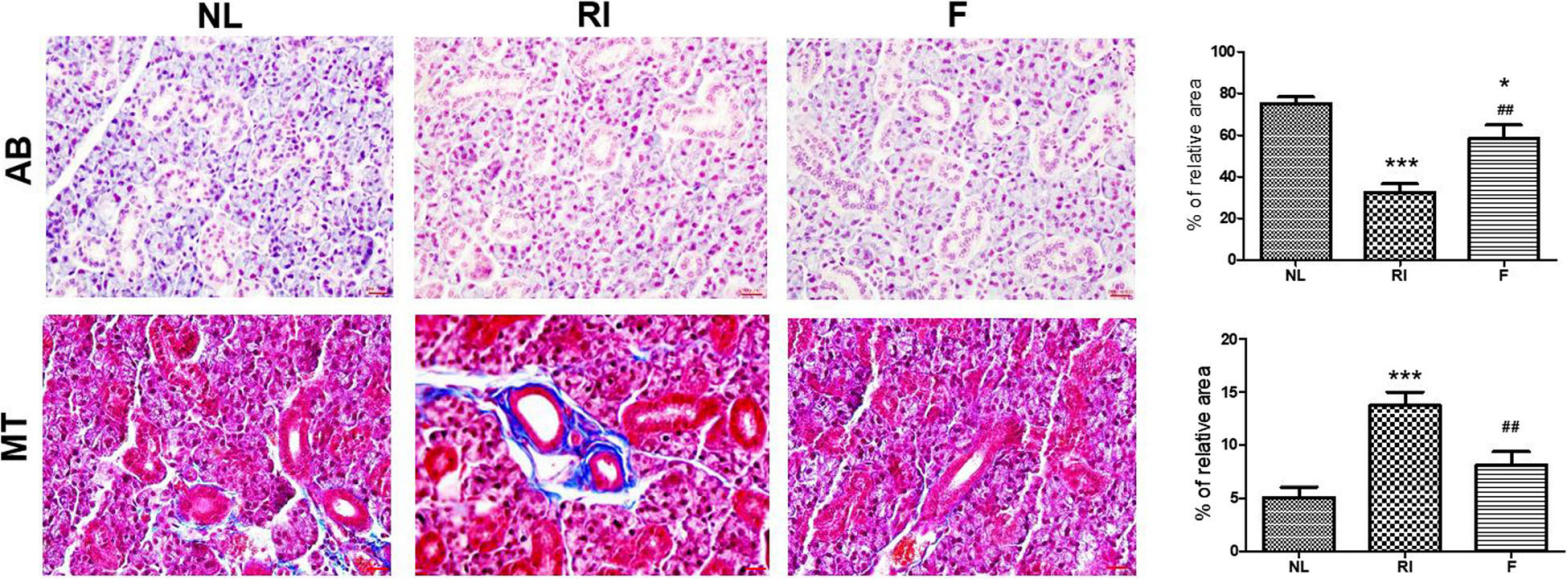
Histological analysis of salivary glands at 12 weeks post-RI treatment. In Alcian blue (AB) staining, the fucoidan group had more mucin-containing parenchymal areas than the RI-exposed group (RI group). Masson’s trichrome (MT) staining showed that the fucoidan group exhibited less periductal fibrosis than the RI-exposed group. Statistical analyses were conducted using the Kruskal-Wallis test and the Dunn’s post hoc multiple comparison test. Each bar shows the mean ± SD; ^*^, compared to the normal control group; ^#^, compared to the RI group. ^*^p < 0.05, ^***^p < 0.001, ^##^*p* < 0.01, n = 4 mice in all groups; Bar size; 20 μm). NL, normal control; RI, RI-exposed group; F, administration of fucoidan before RI exposure

Histological changes in the SGs were visualized by H & E, AB and MT staining at 12 weeks post-RI. A morphometric analysis of the AB stain, which represents mucin density, showed that mucin production decreased in the RI group (32.6 ± 3.9; mean ± SD) compared to the normal group (75.4 ± 2.9; mean ± SD). However, mucin was significantly increased in the fucoidan group (58.6 ± 6.3; mean ± SD) relative to the RI group (Fig. 2, *p* < 0.05). MT staining, which examines fibrosis, showed the highest degree of fibrosis in the RI group (13.8 ± 1.3; mean ± SD) relative to the normal group (5.1 ± 1.0; mean ± SD), whereas the fucoidan group (8.1 ± 1.2; mean ± SD) showed lower levels of fibrosis compared to the RI group (Fig. 2, *p* < 0.05).

**Fig. 3:**
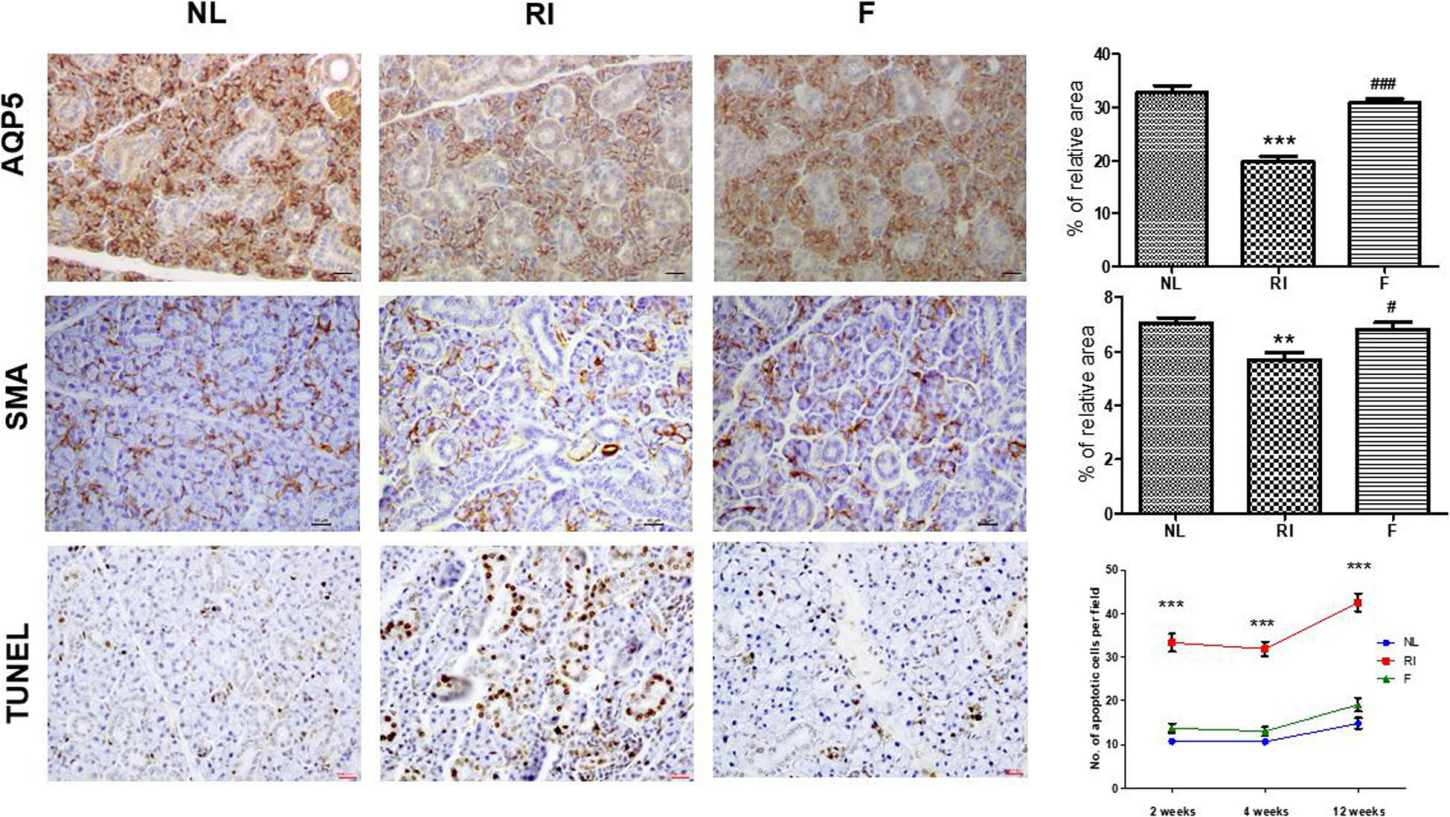
Immunohistochemistry and TUNEL assay of salivary glands at 12 weeks post-RI treatment. Representative immunohistochemical images show salivary epithelial cells (aquaporins 5 (AQP5) staining) and myoepithelial cells (α-smooth muscle actin (α-SMA) staining). The expression level of AQP5 and α-SMA was lower in the radioiodine-exposed group (RI group) than in the normal group. Treatment with fucoidan increased the staining intensity of these cells relative to the RI group. The total number of TUNEL-positive cells was significantly reduced in the fucoidan group as compared with the RI group at 2, 4, and 12 weeks post RI. Statistical analyses were conducted using the Kruskal-Wallis test and the Dunn’s post hoc multiple comparison test. Each bar shows the mean ± SD; ^*^, compared to the normal control group; ^#^, compared to the RI group. ^**^p < 0.01, ^***^p < 0.001, ^#^*p* < 0.05, ^###^*p* < 0.001, n = 4 mice in all groups; Bar size; 20 μm. NL, normal control; RI, RI-exposed group; F, administration of fucoidan before RI exposure

The cytoprotective effects of fucoidan on salivary epithelial and myoepithelial cells were assessed by immunohistochemical staining. The expression of AQP5 (a maker of salivary epithelial cells) and α-SMA (a marker of myoepithelial cells) decreased in the RI group (19.7 ± 1.0, 5.69 ± 0.3; mean ± SD, AQP5 and α-SMA, respectively) compared with the normal group (32.7 ± 1.4, 7.5 ± 0.2; mean ± SD, AQP5 and α-SMA, respectively) (Fig. 3, *p* < 0.05). Treatment with fucoidan (30.8 ± 0.7, 6.8 ± 0.3; mean ± SD, AQP5 and α-SMA, respectively) increased the staining intensity of these cells relative to the RI group, suggesting that fucoidan can preserve salivary epithelial and myoepithelial cells against RI-induced cell damage (Fig. 3, *p* < 0.05). TUNEL assays showed that the number of TUNEL-positive cells was significantly higher in the RI group (33.3 ± 2.1, 31.9 ± 1.7, 42.6 ± 2.1; mean ± SD at 2, 4, 12 weeks respectively) and significantly lower in the fucoidan group (13.8 ± 1.1, 13.1 ± 1.0, 19.1 ± 1.5; mean ± SD at 2, 4, 12 weeks respectively) at 2, 4, and 12 weeks post-RI as compared to the control group (10.8 ± 0.7, 10.7 ± 0.6, 14.9 ± 1.2; mean ± SD at 2, 4, 12 weeks respectively) (Fig. 3, *p* < 0.05).

### Dynamics of ^99m^Tc pertechnetate uptake and excretion

**Fig. 4:**
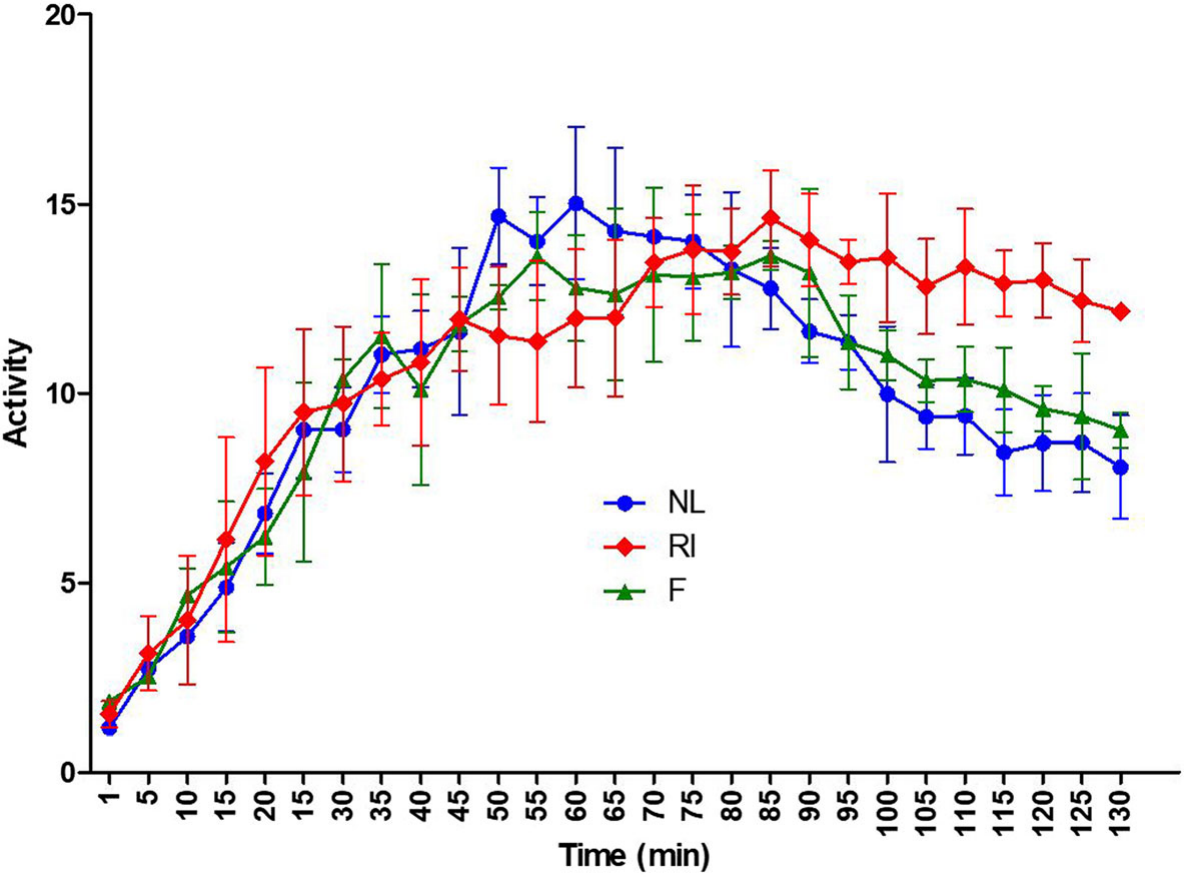
Dynamics of ^99m^Tc pertechnetate uptake and excretion. ^99mc^Tc pertechnetate excretion was lower in the radioiodine (RI)-exposed group (RI group) than in the other groups, whereas ^99mc^Tc pertechnetate excretion in the fucoidan groups was similar to that observed in the normal group. NL, normal control; RI, RI-exposed group; F, administration of fucoidan before RI exposure

The ability of ^99m^Tc pertechnetate excretion was decreased in the RI group at 12 weeks post-RI, but the ability of excretion in the fucoidan group were recovered like that in the normal control group (Fig. 4).

## Discussion

Based on our results, our study suggests, for the first time, that fucoidan administration can attenuate RI-induced SG damage. SG dysfunction is one of the most serious complications of RI ablation therapy and is a significant impairment to a patient’s quality of life.

Early stage damage induced by RI is characterized by painful swelling of the SGs. This swelling is related to poor oral intake and weight loss. This phenomenon was observed in our study, with significant reductions in body weights in RI treated groups. In the latter stages of RI treatment, SG function continues to worsen and patients complain of dry mouth and significant discomfort. Again, our results support these findings and show that RI treatment leads to significant reductions in salivary flow rates and increased lag times. These functional parameters were improved in the fucoidan-treated group.

Acinar atrophy, inflammation, low mucin stained parenchyma, and ductal fibrosis are known to be the main histopathological features of RI-induced SG damage [13]. In our study, mice pretreated with fucoidan showed higher areas of mucin-containing paranchyma than mice of the RI group. Mucin is an important component of mastication, speech, and swallowing. The fucoidan group also exhibited less periductal fibrosis than the RI group. Fibrosis is a factor that leads to a secretion disorder by inducing ductal stenosis. Our results showed that the histologic characteristics were improved in the fucoidan-treated group relative to the RI group.

RI destroys SGs mainly by damaging cellular components and initiating apoptosis. Immunohistochemical staining showed that the expression of AQP5 and α-SMA was higher in the fucoidan group as compared to the RI group. These findings suggest that fucoidan can preserve salivary epithelial and myoepithelial cells against RI-induced cell damage. Also, TUNEL assays showed that the number of TUNEL-positive cells was significantly lower in the fucoidan group than in the RI group at 2, 4, and 12 weeks post-RI. Han et al. [14] reported that fucoidan inhibits apoptosis-associated proteins and regulates cellular ROS levels. Our findings are consistent with the results of another study, and the results from the fucoidan group could indicate improved gland function. SG ductal dysfunction is recognized as the main feature of RI-induced salivary damage. SPECT is an excellent method to evaluate ductal SG function. Our study confirmed that ^99m^Tc pertechnetate excretion was markedly lower in the RI group, but levels of excretion in the fucoidan group were similar to that in the normal control group.

Intracellular mediator such as inflammatory cytokines induces free radical production and some other signaling pathways that extend the acute reaction of normal tissues. It has a important role in late effects of radiotherapy such as fibrosis, tissue inflammation. [15] So, modulation of intracellular mediators is a potential strategy for mitigation of possible radiation damage. The strategy for the mitigation of radiation injury is administration of a protective agent such as antioxidants [15, 16]. Many natural products which have antioxidant properties have been studied to evaluate their preventive abilities against radiation damage [17, 18]. We reported that natural products, epigallocatechin-3-gallate (EGCG) and ginseng, had a protective effect against RI-induced damage of SGs [7, 19]. Fucoidan is a natural sulfated polysaccharide that exists mainly in the cell wall matrix of various species of brown seaweed and has been widely investigated as an antioxidant, anticancer, and anti-inflammatory agent [20–22]. Moreover, numerous studies have found that fucoidan is protective against tissue damage. Zhang et al. [23] reported that fucoidan was protective against hypoxia-induced cardiomyocyte apoptosis, and the mechanism might involve protecting the cells from oxidative injury. Rhee et al. [24] also reported that fucoidan had protective effects on the radiation-induced damage of blood cells. In our study, we found that fucoidan was effective at protecting against SG damage following RI treatment. Fucoidan administration also prevented the loss of body weight induced by RI. SG functional parameters, such as salivary lag times, salivary flow rate, and the SPECT excretion pattern were also improved by fucoidan administration. Also, histological analysis of SGs revealed that fucoidan-treated mice showed similar features to the control mice, and preserved acinar and myoepithelial cells. Inflammatory responses induce free radical production and influence non-irradiated SGs through a mechanism named bystander effect [25]. Regulating the inflammatory mediators could be a main mechanism for reduction of SGs injury [26]. Yu et. Al. [27] reported fucoidan changes the expression patterns of inflammatory cytokines and it attenuate tissue inflammation and fibrosis. Fucoidan-dependent effects are thought to be caused by its scavenging action on ROS and a modulation of inflammation mediator and apoptosis as a radiation mitigator [23, 24, 27].

This study has some limitations. A fucoidan-treated control group and amifostine-treated positive control group were not included in this study. Also, further studies are needed to clarify the mechanism of action responsible for the protection fucoidan affords. Fucoidan can inhibit cell growth, induce apoptosis and suppress angiogenesis of thyroid cancer cells [28]. This finding suggests that fucoidan administration could selectively protect SGs against RI-induced damage while not having a negative effect on the treatment of thyroid cancer. Additional studies are needed to determine whether fucoidan protects thyroid tumor cells in the process of RI exposure.

## Conclusions

Our study shows that fucoidan administration prior to RI exposure attenuates RI induced SG damage in mice. We believe that fucoidan should be considered as a candidate for preventing SG dysfunction in thyroid cancer patients with RI therapy.

## Data Availability

Not applicable.

## Abbreviations

AB: Alcian blue
AQP5: Aquaporins 5
H&E: Hematoxylin & Eosin
MT: Masson’s trichrome
RI: Radioiodine
SD: Standard Deviation
SG: Salivary gland
SMA: Smooth muscle actin
SPECT: Single photon emission computed tomography
Tc: Technetium
TUNEL: Terminal deoxynucleotidyl transferase biotin-dUDP nick-end labeling
